# Editorial: Angiogenesis blockade for the treatment of gastrointestinal cancer

**DOI:** 10.3389/fonc.2023.1147849

**Published:** 2023-02-01

**Authors:** Alessandro Passardi, Alessandro Bittoni, Zhigang Bai, Zhongtao Zhang, Cornelis Sier, Yulong He, Endrit Shahini, Antonio Giovanni Solimando

**Affiliations:** ^1^Department of Medical Oncology, IRCCS Istituto Romagnolo per lo Studio dei Tumori (IRST) “Dino Amadori”, Meldola, Italy; ^2^Department of General Surgery, Beijing Friendship Hospital, Capital Medical University and National Clinical Research Center for Digestive Diseases, Beijing, China; ^3^Department of Surgery, Leiden University Medical Center, Leiden, Netherlands; ^4^Cyrus Tang Hematology Center, Collaborative Innovation Center of Hematology, National Clinical Research Center for Hematologic Diseases, State Key Laboratory of Radiation Medicine and Protection, Cam-Su Genomic Resources Center, Soochow University, Suzhou, China; ^5^Gastroenterology Unit, National Institute of Gastroenterology - IRCCS “Saverio de Bellis”, Castellana Grotte, Bari, Italy; ^6^Guido Baccelli Unit of Internal Medicine, Department of Precision and Regenerative Medicine and Ionian Area - (DiMePRe-J), University of Bari “A. Moro”, Bari, Italy

**Keywords:** angiogenesis, gastrointestinal cancer, ramucirumab, anti-angiogenic therapies, apatinib

Angiogenesis is defined as a process of new blood vessel formation from pre-existing vessels. Since the concept of anti-angiogenesis for the treatment of cancer was proposed in the 1970’s, tremendous effort has been invested in the field of vascular research. This has led to the development of numerous agents targeting angiogenesis, with over a dozen of anti-angiogenic drugs approved in clinic application and more in the pipeline of clinical trials (1).

The role of angiogenesis in gastrointestinal tumours is well known and anti-angiogenic agents are widely used in combination with chemotherapy with improved survival outcomes, most notably in colorectal cancer and hepatocellular carcinoma. The review edited by Gonzalez and colleagues focuses on the clinical evidence of efficacy, the ongoing clinical trials and the preclinical rationale underlying new combinations, especially with immunotherapy (Gonzalez et al.). Despite strong preclinical rationale and promising preliminary results in early clinical trials, anti-angiogenic therapies failed to revolutionize anti-cancer treatment in these tumour types. In this context, a greater knowledge of the mechanisms underlying primary and acquired resistance is an essential premise to improve treatment efficacy (Schiffmann et al.). A promising approach to overcome resistance is the use of nanomedicine. In fact, nanoparticles have shown significant advantages as anti-angiogenic drugs favouring targeted delivery, controlled release, prolonged half-life, and increased bioavailability (Yang et al.).

Angiogenesis inhibition is expected to be a promising therapeutic strategy in advanced gastric cancer (AGC). Several trials have been conducted to evaluate the efficacy of anti-angiogenic agents in metastatic disease, but with conflicting results. The most critical efficacy data were reported with ramucirumab, a fully humanized monoclonal antibody directed against the vascular endothelial growth factor receptor-2 (VEGFR2). Ramucirumab in combination with paclitaxel significantly improved overall survival compared to placebo plus paclitaxel in patients with advanced gastric or gastro-oesophageal junction (GEJ) adenocarcinoma in the global phase 3 RAINBOW study (2). Similarly in the RAINBOW-Asia, a study with a similar design conducted in Asian patients, the median progression-free survival was higher in the ramucirumab plus paclitaxel group than placebo plus paclitaxel group. However the median overall survival was similar (3). On the other hand, trials testing other anti-angiogenic agents and early phase randomized trials (in both neoadjuvant and first-line settings) have shown negative results. Moreover, the lack of predictive biomarkers does not permit to select patients more likely to benefit from an anti-angiogenic approach (Salati et al.). A prospective study investigated the circulating angiogenic biomarkers’ predictive role in thirty-five advanced AGC patients receiving ramucirumab and paclitaxel as second-line therapy (D’Alessandro et al.). Results showed that a greater decrease in VEGFC and Ang2 levels measured at the beginning of the third cycle of therapy compared to baseline corresponded to a lower risk of progression and therefore a longer progression-free survival. Interestingly, the study also showed an increase in VEGFC and Ang2 at the progression time, suggesting the activation of alternative pathways such as VEGFC/VEGFR3 and Ang2/Tie2 and supporting the rationale for dual inhibition of Ang2 and VEGRs.

Recent data suggest that inhibition of angiogenesis may also be helpful in preventing the occurrence and progression of gastric cancer precursor lesions (GPL). GPL refers to pathological changes of the gastric mucosa, including atrophic gastritis, intestinal metaplasia and dysplasia associated with the development of gastric cancer. In a preclinical study, Gao et al. investigated the activity of Atractylenolide III (AT-III), the main bioactive component of the traditional Chinese medicinal herb Atractylodes macrocephala, on GPL angiogenesis and expression of angiogenesis related factors. The authors found that AT-III reduced microvessels density and attenuated early angiogenesis in GPL rat models. Moreover, they showed a reduction of HIF-1α and VEGF-A, two important angiogenic markers, in GPL tissues after AT-III treatment and downregulation of DLL4, a component of the Notch signalling pathway involved in angiogenesis. These exciting results suggest a possible role for inhibition of angiogenesis with AT-III in treating gastric cancer precursor lesions, reducing the incidence and mortality of gastric cancer.

Apatinib is the first anti-angiogenic drug approved for treating advanced or metastatic gastric adenocarcinoma in China, where ramucirumab is unavailable. It is recommended in the third line setting, and despite small evidence of efficacy also as second line (Fu et al.). A recent trial explored a new scoring system calculated by combining systemic immune-inflammation index (SII) and prognostic nutritional index (PNI) as a predictor of efficacy in patients treated with intraperitoneal and systemic paclitaxel combined with Apatinib conversion therapy for gastric cancer with positive peritoneal cytology (Ding et al.). The prognosis of patients with high SII-PNI score was significantly worse and multivariate analyses confirmed the score as an independent prognostic factor for both overall survival and progression-free survival.

The phosphatidylinositol-3 kinase (PI3K) signalling pathway plays an essential role in cancer cell survival, angiogenesis and metastasis in several types of tumours, including colorectal cancer (CRC) (4). Recently, inhibition of the PI3k/Akt/mTOR pathway has become a promising therapeutic strategy in CRC patients with some encouraging preliminary results (5). An interesting study by Qin et al. sheds some light on the role of targeting PI3K in colorectal cancer and offers insights into PI3K inhibition biological effects. The authors evaluated ZDQ-0620, a novel pan-PI3K inhibitor, on human CRC cell lines demonstrating a significant activity in terms of inhibition of proliferation, migration and invasion. In addition, it was shown that ZDQ-0620 can significantly suppress angiogenesis through the inhibition of endothelial cell tube formation and vasculogenic mimicry. These data reinforce the evidence of an association between the PI3k/Akt/mTOR pathway and the VEGF-induced endothelial signalling, supporting the rationale for combinatorial PI3K and VEGF inhibition strategies in colorectal cancer, as already studied in other malignancies (6).

Inhibition of angiogenesis is a cornerstone of the treatment of neuroendocrine neoplasms (NENs). In their paper, Lauricella et al. provide an overview of the main molecular events driving angiogenesis in NENs and molecular mechanisms of resistance to anti-angiogenic drugs in these malignancies. In addition, authors discuss the results of clinical trials of several anti-angiogenic agents, including novel compounds such as the HIF-2a inhibitor belzutifan, and different combinatorial treatment, including association of anti-angiogenic agent to immunotherapy or mTOR inhibitors, offering a perspective about present and future treatment of NENs.

## Author contributions

AP, AB and AS wrote the first draft of the manuscript, all the authors contributed to manuscript revision, read, and approved the submitted version.
